# Biomedical Cloud Computing With Amazon Web Services

**DOI:** 10.1371/journal.pcbi.1002147

**Published:** 2011-08-25

**Authors:** Vincent A. Fusaro, Prasad Patil, Erik Gafni, Dennis P. Wall, Peter J. Tonellato

**Affiliations:** 1 Center for Biomedical Informatics, Harvard Medical School, Boston, Massachusetts, United States of America; 2 Department of Pathology, Beth Israel Deaconess Medical Center, Boston, Massachusetts, United States of America; Whitehead Institute, United States of America

## Introduction

Biomedical research in the post-genome era is intensely data-driven and increasingly more integrative as new technologies are introduced, such as next- or third-generation sequencing, mass spectrometry, and imaging to identify novel biological insights. The volume and complexity of biomedical data is increasing exponentially as faster high-throughput machines are introduced. As a result, many research institutes, biotech companies, pharmaceutical companies, and computational labs are considering cloud computing as a cost-effective alternative to process and store this vast amount of data. Efforts in next-generation sequencing (NGS) [1]–[3], comparative genomics [4], and proteomics [5] have already successfully incorporated the cloud to expedite their data processing. The challenge remains to decide how to best take advantage of the flexibility of cloud computing to conduct these and other analyses. The purpose of this overview is three-fold: 1) introduce biomedical cloud computing, 2) provide a concrete methodology detailing how projects are developed on the cloud, and 3) demonstrate cloud computing costs. We assume the reader has a basic understanding of UNIX.

There are multiple cloud providers, both commercial and open source, including Amazon Web Services (AWS), Rackspace, GoGrid, Nimbus, and Eucalyptus, each contributing to the popularity and globalization of cloud computing. For the purposes of this guide, we focus on the use of AWS as the cloud computing platform and adopt the definition of Vaquero, who states that the cloud is “a large pool of easily usable and accessible virtualized resources (such as hardware, development platforms, and/or services). These resources can be dynamically re-configured to adjust to variable load (scale), allowing for optimum resource utilization” [6]. Cloud computing is ideal for projects that require periodic computational bursts, rapid prototyping, or fast turnaround time. Furthermore, the cloud is an attractive alternative to the limitations imposed by a local computing environment such as long job queues, unsupported software, or limited server resources. One key difference between traditional server-based (grid) computing and the cloud is virtualization technology, which enables the partitioning of a server's hardware resources into multiple “instances”, each running its own operating system in isolation from the other instances. In practice, the virtualized instance appears to the user to be an entirely separate computer, even though the virtual instance may share a combination of independent central processing units (CPUs), memory, and storage devices with other virtualized instances. An example of this is running Windows on Mac OS using Parallels or VMware. The economic model for cloud computing is another key difference, where you only pay for what you use, much like electricity or water. In essence, cloud computing is a commodity service that can provide on-demand access to a computational infrastructure and avoids the fixed cost of capital investments in computing hardware, computing maintenance, and personnel.

## Amazon Web Services

AWS provides the necessary computing environment, including CPUs, storage, memory (RAM), networking, and operating system, and is an example of “infrastructure as a service” (IaaS). IaaS is popular with computational biologists because it offers more flexibility for designing projects ad hoc. The majority of computational projects will make use of three AWS products (see “Get Started with EC2” at http://docs.amazonwebservices.com/AWSEC2/2009-11-30/GettingStartedGuide/): Elastic Compute Cloud (EC2), Elastic Block Storage (EBS), and Simple Storage Service (S3). For additional AWS products beyond the scope of this overview, we refer the reader to the AWS Web site (http://aws.amazon.com/). EC2 contains a variety of user selectable instance types that range in computing power and cost (Table 1). An instance boots within a few minutes and the user is given root or administrator access. An EBS volume is a storage device that can be attached to a running instance, similar to a USB thumb drive, and currently ranges in size from 1 GB to 1 TB. EBS volumes are redundantly backed up and offer approximately 99.7% durability, but they can be further backed up to S3 by taking a snapshot of the drive (an incremental backup). S3 is an extremely reliable persistent storage system that also makes data readily available over the Internet. To ensure reliability, the file system of S3 is composed of “buckets” that are geographically distributed across Amazon's multiple data centers so that each file is backed up in several locations. Thus, AWS is able to offer 99.999999999% durability and 99.99% availability for file objects. By default all buckets are marked as private; however, Amazon and other institutions are making large datasets available over S3 via public buckets (http://aws.amazon.com/publicdatasets/). Access to all of AWS's services can be done using either a Web-based console, for beginners, or through the command line using an AWS-specific application programming interface (API), for advanced users. AWS costs are generally based either on an hourly rate or amount of data transferred or stored or other services used (Table 1).

**Table 1:** A summary of AWS pricing for basic computation, storage, and data transfer.

Resource Type	Example Use	AWS Service	Service Unit	CPUs (#xGHz)^1^	Memory (GB)	Cost ($/Hr)^2^
**Computation**	Running a 51-node cluster (50 m2.2xlarge workers and one m1.small master) for 8 hours costs **$400.68**.	EC2	t1.micro	2×1	0.6	0.020
			m1.small	1×1	1.7	0.085
			m1.large	2×2	7.5	0.340
			m1.xlarge	4×2	15	0.680
			c1.medium	2×2.5	1.7	0.170
			c1.xlarge	8×2.5	7	0.680
			m2.xlarge	2×3.25	17.1	0.500
			m2.2xlarge	4×3.25	34.2	1.000
			m2.4xlarge	8×3.25	68.4	2.000
			cc1.4xlarge	2×(4×4.19)1a	23	1.600
			cg1.4xlarge	2×(4×4.19)1b	22	2.100
**Resource Type**	**Example Use**	**AWS Service**	**Service Unit**	**Size**	**Tiers** **(per Month)**	**Cost ($/GB/Month)**
**Storage**	Maintaining 5 buckets (4×50 GB data files and 1×30 GB results) for 4 months costs **$32.20**.	S3	S3 Bucket	Virtually unlimited	First 1 TB	0.140
					Next 49 TB	0.125
					Next 450 TB	0.110
					Next 500 TB	0.095
					Next 4000 TB	0.080
					5,000+ TB	0.055
	Attaching 3 EBS volumes to an instance (2×100 GB and 1×30 GB) for 1 month costs **$23.00.**	EBS	EBS Volume	Up to 1 TB	N/A	0.100
**Resource Type**	**Example Use**	**AWS Service**	**Service Unit**	**Data Transfer Type**		**Cost ($/GB/Month)**
**Data Transfer**	Uploading 230 GB of data to S3 and downloading 12 GB of results costs **$25.00**.	EC2, S3	I/O	Data IN		0.000 (free)
				Data OUT First 1 GB		0.000 (free)
				Data OUT Next 10 TB		0.120
				Data OUT Next 40 TB		0.090
				Data OUT Next 100 TB		0.070
				Data OUT 150 TB+		0.050
				Between AWS Services^3^		0.000
		EBS	I/O^4^	Per 1 m I/O Requests		0.100
		S3	API Request^4^	PUT, COPY, POST, LIST Request		0.01 (per 1,000)
				GET Request		0.01 (per 10,000)

Prices are current as of 7/05/11.

1 CPUs are in terms of a 1-GHz Opteron 2007 processor, unless otherwise noted. For example, a machine with four 1-GHz processors would be listed as 4×1.

1a CPU is a quad-core Xeon X5570, i.e., two quad-core CPUs, where each core is 4.19 GHz.

1b CPU is a quad-core Xeon X5570, and instance includes two NVIDIA Tesla "Fermi" M2050 GPUs.

2 Costs reflect standard EC2 use with Linux OS. Costs increase when using Windows and decrease when using Reserved Instances (up-front payment) or Spot Instances (user-specified price on unused EC2 capacity).

3 Within same AWS availability region (e.g., AWS US-East).

4 Request costs are more difficult to estimate, and are usually more pertinent when databases and other similar services are involved. Programs like IOSTAT can be used to estimate EBS requests.

## Security in the Cloud

Before beginning to use the cloud, it is important to understand the basic best practices for cost control and data security (for more detailed information, see http://media.amazonwebservices.com/pdf/AWS_Security_Whitepaper.pdf).

### Use Public/Private Key Pair

In this cryptographic scheme, a pair of keys is constructed, a public encryption key and a private decryption key. The public key is made available to anyone, but the data encrypted by it can only be decrypted by its paired private key. The private key should never be shared because it represents digital proof of the user's identity. Public/private keys provide a more secure login authentication method than usernames and passwords. To grant someone access to an instance, the public key must be copied into the list of authorized keys.

### Access an Instance Using a Secure Connection

For security of data and encryption of data transfers, it is imperative that access to an instance is via a secure protocol such as Secure Shell (ssh) or Secure Copy (scp). Both the AWS Web console and command line tools provide a simple interface to generate key pairs when launching an instance. The public key is automatically installed onto an instance, and the private key can then be used on a computer that will ssh into that instance.

### Create Restricted User Accounts

Recently, AWS introduced Identity and Access Management (IAM) to offer greater control and management of multiple users. Each user has their own set of security credentials to access cloud resources, eliminating the need to share login information and keys for the master AWS account owner. This is important because the master account contains the personal billing information, which, for obvious reasons, should not be accessible to all users. IAM can restrict services based on specific users or group policies. For example, it is possible to restrict a user to a specific S3 bucket between 9 A.M. and 5 P.M. from a specific IP address.

### Control Access Using Firewalls

A security group defines a set of rules that govern how traffic (data or communication) reaches the AWS instance. By default, the security group restricts all inbound traffic, allows all outbound traffic, and allows other instances within the group to communicate. These rules can be completely customized; for example, it is possible to restrict access to a specific IP address on a specific port address and not allow that instance to communicate with other instances within the AWS account.

### Additional Security

All security keys should be replaced with new ones every 30–90 days. Installing regular software updates is essential to protect the operating system and third-party software from vulnerabilities. There are many additional security features such as private clouds (http://aws.amazon.com/vpc/), encrypted file systems, and encrypted data volumes that may be used by those who have security needs beyond these basic best practices.

## Prototyping and Development

An often-overlooked aspect of cloud computing is running only a single instance where scalability is not a requirement. Simple tasks such as making certain programs run faster by using a faster CPU, increasing the memory, prototyping, or even small Web applications are easily addressed by using a more powerful single instance (Table 1). In some situations, such as daily analysis or constant development, the cost is low enough to leave those instances running on a continuous basis.

It is essential to have a clear understanding of the technical requirements of the project in order to select the proper cloud resources. There are three basic criteria to consider for a given project to accurately estimate the cost: hardware, data, and analysis time. First, estimate the amount of memory, disk space, and CPUs needed for the computational task. For existing software, this is often found in the user documentation. For new code development, it may require an iterative process to determine the most suitable instance type. The UNIX “top” command is a good way to check the resource utilization. Second, estimate the amount of data required for analysis. AWS charges per gigabyte to transfer data out of their cloud (data transfer in is now free) and also per gigabyte for persistent storage (Table 1). It is easy to inadvertently incur unnecessary transfer costs as a consequence. Third, estimate the amount of time it will take to complete the analysis, because AWS begins charging for an instance the moment it is launched. Importantly, there are no cost savings by running fewer than the maximum number of instances necessary to complete an analysis because the cost is based on the amount of time an instance is running. For example, using half the number of instances, the job will take twice as long to finish and will end up costing the same amount based on instance runtime—meaning, there is no reason to wait longer for your results than you have to.

## Developing a Scalable Computing Environment

A large-scale computing environment that scales up or down in response to computational demand is the most commonly perceived use of cloud computing because it takes full advantage of rapid replication and linear scaling of cheap commodity compute cycles. However, it is important to remember that the cloud does not “magically” enable programs to run more efficiently or in parallel (unless the code was already written that way). Instead, it requires an understanding of how to connect multiple instances together to form a cluster and knowledge of how to divide a computational task into sub-components that can run simultaneously. Until recently, cluster creation was onerous, requiring substantial amounts of customized solutions handled best by an expert in systems administration and computer science. Fortunately, new advances in open source cluster management software such as StarCluster (http://web.mit.edu/stardev/cluster/), Boto (http://code.google.com/p/boto/), Condor (http://www.cs.wisc.edu/condor/), and Hadoop (http://hadoop.apache.org/mapreduce/) are making cluster creation, termination, and job queuing more automated and accessible to bioinformatics specialists with perhaps only a limited understanding of systems administration and architecture. Within AWS, there is also the option to use Elastic Map Reduce (AWS's implementation of Hadoop) or high performance computing instances (http://aws.amazon.com/ec2/hpc-applications/) for an additional cost per instance. Note that an important consideration for a large cluster is to shut down instances when the number of CPUs is greater than the number of jobs. This will reduce the amount of money spent on idle CPU time, which can be substantial for hundreds or thousands of CPUs [7].

Broadly speaking, scalable computing can be divided into data-intensive distributed applications, of which Hadoop is the prime example, and batch computing, which includes StarCluster and Condor. Hadoop is an open source Java software framework that is composed of two key services: reliable data storage called the Hadoop Distributed File System (HDFS) and a parallel computing technique called MapReduce [8], which was developed by Google to take advantage of commodity computers [9]. Programs must be specifically written using the MapReduce parallel programming model. Although there are many successful applications of Hadoop, including processing NGS data [1], not all programs fit this model and learning the Hadoop framework can be challenging. Batch computing is a simpler programming model that operates by creating a set of tasks that are processed independently and is typical of institutional clusters that run LSF or Sun Grid Engine. StarCluster was created to simplify the cluster creation, management, and job scheduling on AWS. Because many biomedical computing projects are easily divided into independent tasks and using Hadoop is more technical, we will demonstrate the use of StarCluster in the case study example.

## Case Study: Creating a Whole Genome Mapping Computational Framework

To put the previous concepts into practice, we will walk through the analysis of a large amount of NGS data. Specifically, we detail the creation of a pipeline to process an entire human genome's worth of NGS reads using a short read mapping algorithm. We use the ∼4 billion paired 35-base reads sequenced from a Yoruba African male [10] to test the pipeline. For this case study, we selected the open source sequence alignment tool MAQ [11] to map the reads and identify the variants. Although there are newer and more efficient alignment algorithms, MAQ is a good example of software that was not initially designed to run in parallel and is typical of most bioinformatics software. The African genome read set is 370 GB with individual files containing nearly 7 million reads each. Computation time for just one of the 303 read file pairs typically ranges from 4 to 12 hours, and files with more ambiguous reads may require over a day to be fully mapped to the reference human genome. The cloud is an ideal platform for processing this dataset because the computational resources required to run these intensive mapping steps can be allocated quickly and easily, and because mapping short reads to a reference genome is a task that is readily distributed over a compute cluster.

## Prototyping and Development (Total Cost: $3.85)

**Figure 1:**
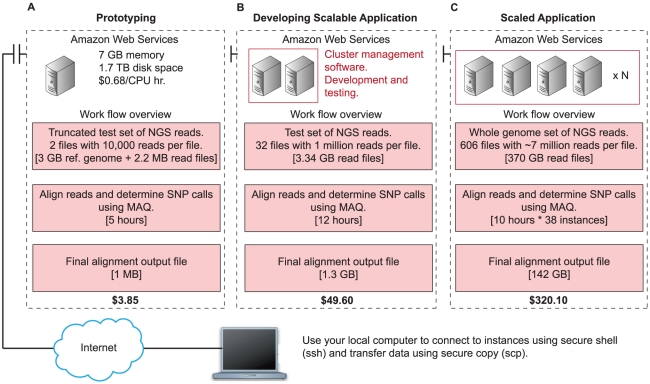
Step-wise framework for creating a scalable NGS computing application. Using your local computer, ssh into an instance running in AWS. The costs are representative of actual development time, data transfer into and out of the cloud, and the compute time using AWS (Table 1). The costs presented may vary, as AWS frequently updates their pricing structure. (A) An additional 3 hours were included for installing programs and testing the instance for the prototyping phase. (B) An additional 2 hours were included in developing the scalable application to learn how to use the cluster management software. (C) For the final scaled application, we used a 38-instance cluster.

The NGS mapping example begins by prototyping and testing the whole-genome mapping pipeline (Figure 1A). At this stage, we are interested in testing the mechanics of launching a single instance, installing MAQ, and processing two truncated files (10,000 reads per file). Based on the technical requirements specified in the section above and referring to the MAQ reference manual, we learn that mapping 1 million paired reads takes on average 10 hours and uses 800 MB of memory. Therefore, a single extra-large Linux instance (7 GB memory and eight CPUs) from AWS priced at $0.68/hr can easily handle the truncated example files containing 10,000 reads (Table 1). Using the AWS console, we launch a c1.xlarge instance type with the latest stable release of Ubuntu. From the AWS console, we create a 400 GB EBS volume and attach it to the running instance. Once the instance boots, we login via ssh as root administrator using the public IP address (for example, ec2-184-73-252-16.compute-1.amazonaws.com), download and install MAQ, and copy the NCBI reference genome to a directory on the instance using scp or Wget (http://www.gnu.org/software/wget/). Next, we format and mount the EBS volume and create three directories for testing the mapping data—small (two files, 10,000 reads), medium (32 files, 1 M reads), and all (entire genome). Then, we upload the African genome and the smaller testing files into the appropriate directories. Following the MAQ instructions (http://maq.sourceforge.net/maq-man.shtml) and executing the mapping and assembly commands, we learn that it takes 2 hours to analyze one pair of read files from the “small” directory on the EBS volume. However, only one of the possible eight CPUs on the extra-large instance is in use because we only issued one MAQ map command. While we could manually launch eight MAQ commands, a better approach would be to use cluster management software to automatically take advantage of all eight CPUs and include additional instances.

## Developing a Scalable Computing Environment (Total Cost: $49.60)

Next, we will introduce the use of StarCluster to create and manage a small test cluster of two instances. StarCluster is customized for use on AWS and uses the open source version of Sun Grid Engine (http://gridscheduler.sourceforge.net/index.html) to manage batch queuing across distributed systems, along with OpenMPI to manage job distribution and instance communication. The cluster is composed of a master instance, which is responsible for managing a larger set of worker instances. In this example, each worker instance is able to process eight jobs concurrently and will contain the necessary software to run the analysis. In order to get StarCluster running, we need to work through a few steps that involve configuring the StarCluster instance type, setting the proper security, and installing StarCluster on your local computer to remotely create and terminate a cluster.

First, we will configure the StarCluster base instance type or Amazon Machine Image (AMI) with our required software. An AMI packages the operating system, installed programs, and user settings into a binary file that can be launched to exactly replicate an environment. Amazon creates a unique private ID (default) or public ID for each AMI to launch identical instances. We locate the StarCluster AMI through the AWS console under Community AMIs (for example, ami-0af31963), launch it, and attach the previously created EBS volume containing the NGS data to the running instance. Then we install MAQ and any additional processing scripts as before. Next, we need to bundle the instance into an AMI in order to allow StarCluster to launch multiple identical instances. After bundling the AMI (http://docs.amazonwebservices.com/AWSEC2/2011-02-28/UserGuide/), we record the AMI ID—we will use this later in the StarCluster configuration file. We take a snapshot of the EBS volume to back it up in S3. We will use the snapshot ID later in the StarCluster configuration file to allow each instance access to the data.

Second, we follow our best practices and create a new security group and key pair for the cluster. This provides more security control in your AWS account and allows you to easily revoke credentials in the unlikely event that the account is compromised. Should further security be desired, it is straightforward to create a “cluster user” using IAM to further restrict access to S3 accounts or limit the number of instances available to launch.

Third, we install StarCluster on our local computer following their documentation (http://web.mit.edu/stardev/cluster/docs/index.html). The installation package includes the necessary scripts and configuration files to manage a cluster. The configuration file contains the various parameters to specify the cluster creation such as AMI ID, number of instances to launch, AWS account credentials, instance type, key pair, EBS snapshot ID containing the NGS data, and security group.

At this point in our case study, we are interested in testing the scalability of the NGS mapping pipeline by creating a small cluster and confirming that the environment is functioning as expected (Figure 1B). Using StarCluster and the appropriate configuration file, we launch a two-instance cluster from the command line on our local machine. StarCluster returns the IP address of the master instance and from there we can ssh into the instance, verify Sun Grid Engine is running using the command “qhost”, and run a script to launch a set of jobs from the medium directory on the EBS volume using standard Sun Grid Engine options. For this example, a job is defined as mapping each read file to the reference genome. This is an independent task—meaning it does not require additional information from other reads or jobs to be completed successfully. We monitor the job progress using the command “qstat” (http://gridscheduler.sourceforge.net/htmlman/manuals.html). When the jobs are finished, we can save the results on the EBS volume and shut down the cluster.

## Scaled Production Environment (Total Cost: $320.10)

We now expand our case study to the next level of usage, one that best exemplifies the most common conception of cloud computing: a virtually unlimited computational environment, which an analysis task will harness for rapid completion. However, getting to this point requires successful prototyping of an application, namely the prior two stages outlined above, on the cloud and ensuring that your application and pipeline can run on two or more inter-communicating instances.

Returning to our case study, we want to create the environment to process the entire human genome (Figure 1C). The previous discussion laid the foundation for creating a scalable computing environment such that increasing the cluster size is as easy as modifying the StarCluster configuration script—in this case we specify 38 instances (38 * 8 CPUs  = 304 CPU cluster) in the configuration file. After launching the cluster, we update our job submission script to use the appropriate directory on the EBS volume that contains the entire 370 GB read data. We also configure Sun Grid Engine to only allow jobs to run for a maximum of 10 hours in order to manage the cost. If a job does not finish within that time limit, it will automatically be terminated and noted in the log file for future analysis. We save the final alignment results to the EBS volume and copy the file (142 GB) to our local computer and terminate the cluster.

## Summary

In this overview to biomedical computing in the cloud, we discussed two primary ways to use the cloud (a single instance or cluster), provided a detailed example using NGS mapping, and highlighted the associated costs. While many users new to the cloud may assume that entry is as straightforward as uploading an application and selecting an instance type and storage options, we illustrated that there is substantial up-front effort required before an application can make full use of the cloud's vast resources. Our intention was to provide a set of best practices and to illustrate how those apply to a typical application pipeline for biomedical informatics, but also general enough for extrapolation to other types of computational problems. Our mapping example was intended to illustrate how to develop a scalable project and not to compare and contrast alignment algorithms for read mapping and genome assembly. Indeed, with a newer aligner such as Bowtie [9], it is possible to map the entire African genome using one m2.2xlarge instance in 48 hours for a total cost of approximately $48 in computation time. In our example, we were not concerned with data transfer rates, which are heavily influenced by the amount of available bandwidth, connection latency, and network availability. When transferring large amounts of data to the cloud, bandwidth limitations can be a major bottleneck, and in some cases it is more efficient to simply mail a storage device containing the data to AWS (http://aws.amazon.com/importexport/). More information about cloud computing, detailed cost analysis, and security can be found in references [12]–[14].
